# Small medial femoral condyle morphotype is associated with medial compartment degeneration and distinct morphological characteristics: a comparative pilot study

**DOI:** 10.1007/s00167-020-06218-8

**Published:** 2020-08-14

**Authors:** Jonas Grammens, Annemieke Van Haver, Femke Danckaers, Brian Booth, Jan Sijbers, Peter Verdonk

**Affiliations:** 1 Antwerp Surgical Training, Anatomy and Research Centre (ASTARC), University of Antwerp, Universiteitsplein 1, 2610 Wilrijk, Belgium; 2 MoRe Institute, Stevenslei 20, 2100 Deurne, Belgium; 3 imec-VisionLab, Department of Physics, University of Antwerp, Universiteitsplein 1, 2610 Wilrijk, Belgium; 4 Antwerp Orthopaedic Centre Monica Hospitals, Stevenslei 20, 2100 Deurne, Belgium

**Keywords:** Knee joint, Femur, Tibia, Meniscus, Cartilage, Anatomy, 3-D imaging, Osteoarthritis

## Abstract

**Purpose:**

Early-onset degeneration of the knee is linked to genetics, overload, injury, and potentially, knee morphology. The purpose of this study is to explore the characteristics of the small medial femoral condyle, as a distinct knee morphotype, by means of a landmark-based three-dimensional (3D) analysis and statistical parametric mapping.

**Methods:**

Sixteen knees with a small medial femoral condyle (SMC) were selected from a database of patients with distinct knee joint anatomy and 16 gender-matched knees were selected from a control group database. 3D models were generated from the medical imaging. After normalization for size, a set of pre-defined landmark-based parameters was analysed for the femur and tibia. Local shape differences were evaluated by matching all bone surfaces onto each other and comparing the distances to the mean control group bone shape.

**Results:**

The small medial condyle group showed a significant association with medial compartment degeneration and had a 4% and 13% smaller medial condyle anteroposteriorly and mediolaterally, whereas the distal femur was 3% wider mediolaterally. The lateral condyle was 2% smaller anteroposteriorly and 8% wider mediolaterally. The complete tibial plateau was 3% smaller mediolaterally and the medial tibial plateau was 6% smaller.

**Conclusion:**

A new knee morphotype demonstrated an increased risk for medial compartment degeneration and was differentiated from a healthy control group based on the following morphological characteristics: a smaller medial femoral condyle and medial tibial plateau, a wider lateral femoral condyle and a wider distal femur on a smaller tibial plateau. This pilot study suggests a role for the SMC knee morphotype in the multifactorial process of medial compartment degeneration.

**Level of evidence:**

III

## Introduction

The relationship between specific knee morphotypes and pathology has already been investigated for several morphological variations [1, 4, 7, 12, 28, 29, 31]. Implications posed by these distinct morphotypes are of great clinical importance since they may contribute in prevention, lead to a better treatment choice (optimised for each specific patient) and even to new personalised therapies. Based on several morphological studies, it can be concluded that the standard treatment may not meet the needs of certain groups of patients. Furthermore, the huge variability of the coronal alignment, reported in both osteoarthritic and non-osteoarthritic knees suggests a more individualized treatment approach in restoring the functionality of the knee [14, 15, 23]. In addition to the coronal joint line alignment phenotype, the morphotype of the knee joint (shape of the distal femur and proximal tibia) is a complementary concept to describe and investigate anatomical variations in relation to early degeneration of the knee joint; smaller joint contact surfaces may increase the contact stress and might lead to overload.

Though increased shape-related stresses are difficult to investigate in living patients, specific knee morphotypes are already known to be related to certain pathologies. Trochlear dysplasia is characterized by a reduced contact surface area in the patellofemoral joint and is associated with patellar instability and early patellofemoral osteoarthritis [8, 16, 34]. Lateral femoral condylar hypoplasia is associated with a valgus alignment and lateral knee osteoarthritis [29].

In addition to the already documented associations between shape and pathology, a specific knee morphotype, characterized by a smaller medial femoral condyle (SMC), may also result in an increased risk for degenerative changes in the medial compartment of the knee. This morphotype has not been described before and this study might be the first step in exploring the link between bone morphology and early degeneration in the medial compartment.

The aim of this study is to identify knee joint shape differences between SMC knees and a control group and to assess the presence of medial compartment degeneration in both groups. The study hypothesis is that the distal femur and proximal tibia bone shapes of the SMC group differ from those of a control group and that SMC knees demonstrate higher incidence of medial compartment degeneration. To test this hypothesis, two complementary approaches were applied: a landmark-based shape analysis to evaluate a set of pre-defined parameters and a global shape analysis to evaluate local differences. By describing the small medial femoral condyle as a new knee morphotype, this concept can then play an important clinical role in the treatment and the understanding of the multifactorial process of early-onset osteoarthritis (OA) in post-meniscectomised knees.

## Materials and methods

As this is a retrospective study, IRB approval was obtained from the local ethical committee (AZ Monica OG106, study ID number 413) and all persons gave their informed consent prior to their inclusion in the study.

Similar to previous three-dimensional (3D) morphometric studies, a validated landmark-based 3D analysis was performed on the distal femur and proximal tibia to assess the knee joint geometry, including the cartilage [24, 32]. A set of predefined landmarks was manually identified and used to scale the knees isotropically to match the standard size. Additionally, a set of reference planes was also constructed based on these landmarks. This method allows quantification of a predefined set of parameters, which may reveal morphometric differences between control group and SMC knees which have (to our knowledge) not been reported before.

**Fig. 1:**
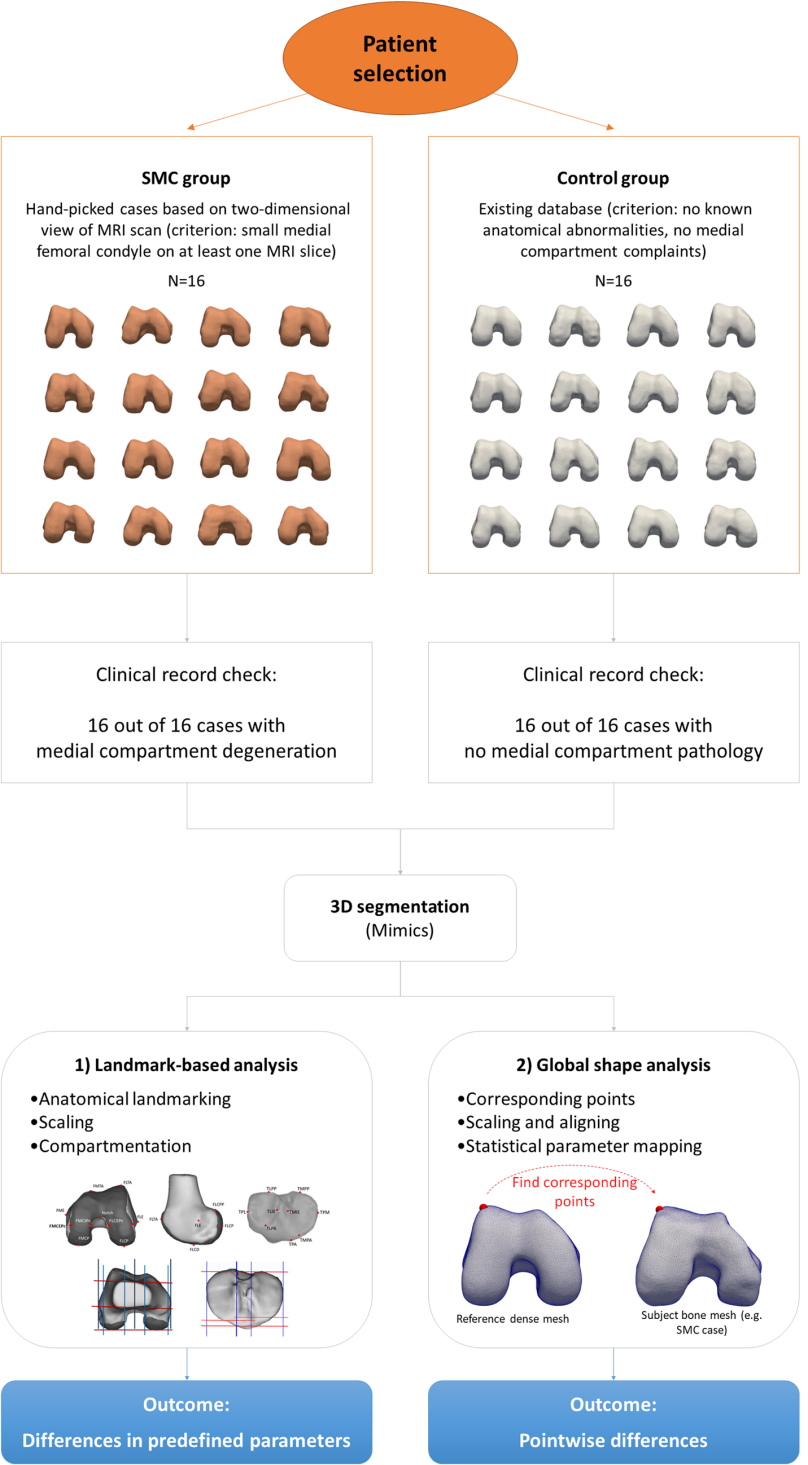
Methodology flowchart. A database of medical imaging was constructed for two groups: the small medial condyle group and a control group. 3D models from tibia and femur were reconstructed from the medical imaging data and postprocessed in a landmark-based analysis and a global shape analysis

Furthermore, a more innovative global shape analysis was performed on the complete bone shape surfaces using statistical parametric mapping. This method is not limited to a number of predefined validated landmarks but includes all points of the bone surfaces and may reveal differences that were not captured in the discrete landmark-based analysis. A flowchart of the study design is presented in Fig. 1.

### Study population

The SMC group included 16 patients (8 females and 8 males; mean age 39 ± 14 years) characterized by a small medial compartment. These patients were selected from a database which was built between 2015 and 2019 by the senior author, who included patients with distinct knee joint anatomy. The selection of the 16 SMC patients was primarily based on the presence of a small medial femoral condyle, observed on at least one slice of the MRI. Second, patients with a short bone stock (caused by limited region of interest) on the MRI and patients who underwent knee surgery prior to the MRI were excluded from this study.

As a control group, 16 patients without anatomic knee abnormalities were selected (8 females and 8 males; mean age 30 ± 9 years). The control group consulted the orthopaedic surgeon for a minor acute trauma and served already as control group in several other studies [24, 32, 33]. Imaging was performed by means of a CT arthrography.

After selecting the patients based on their morphology and the quality of the MRI, the medical history and medical images of the SMC and the control group were inspected in detail to assess medial compartment degeneration.

### Generation and isotropic scaling of 3D computer models

The medical imaging (MRI or CT) was performed in supine position with 0° of knee flexion and the toes pointing straight up. The images were loaded in a 3D image processing software system (Mimics 22.0, Materialise, Haasrode, Belgium) to create 3D models of the knee bones including the cartilage.

Differences in knee size may significantly affect the metric measurements. To avoid this, the 3D models were isotropically resized before the analyses to exclude size differences. A generalized Procrustes transformation of the surface models was applied, using a custom developed code in MATLAB (Matlab 9.6.0, R2019a, Mathworks, Natick, MA), to minimize the pose and size variance between the knees while preserving the shape and underlying proportions of the knees [13]. The applied Procrustes transformation consists of a combination of translation, rotation and isotropic (same amount in the three dimensions) scaling. The standard size was determined by calculating the average femoral shape of the control group. Twelve validated anatomical landmarks, covering the extremes of the distal femur in the anteroposterior (AP), mediolateral (ML) and proximodistal (PD) direction, were defined to calculate a rescaling factor for each knee. This factor was considered as a measure of the femoral size and was also used to isotropically resize the respective tibiae.

### Definition of the landmarks

Anatomical landmarks on the femur and tibia were defined in Mimics (Fig. 1). Both the 3D models and medical images were used to ensure a precise location of the landmarks. The landmarks are described in Appendix A (Table 2) and are identical to those described in our previous studies [24, 32]. Therefore, the ICC of 0.99 and mean error of 1.0 ± 1.5 mm previously reported for this landmarking technique are also applicable to the landmark positions in this study. In addition to the twelve anatomical landmarks which were used to rescale the bone models, eleven other landmarks on the femur and nine landmarks on the tibia were defined to create the reference planes and to measure the morphometric characteristics [24, 32].

### Definition of the reference planes

The reference planes are predefined by the authors in Mimics in the ML, AP and PD direction. An overview table can be found in the “Appendix” (Table 2) of this article. By identifying the landmarks, the reference planes are automatically fitted on the geometry of the distal femur and proximal tibia.

All planes but four are identical to the ones previously described in our other studies [24, 32]. The newly introduced planes are related to the ML width of the medial and lateral condyle.

### Measurements

Based on this set of landmarks and reference planes, 19 morphometric measurements of the 3D models were evaluated as described in the “Appendix” (Table 2) and as summarized in the next paragraph.

For the medial and lateral condyle of the femur, the overall AP size and the AP size of the posterior parts were measured separately. In the ML direction, the total width of the femur, the width of the medial condyle, the lateral condyle, and the notch were measured.

The tibia measurements were performed in a similar way. The AP size of the total tibial plateau, the medial and lateral tibial plateau were measured. In the ML direction, the width of the complete tibial plateau, the medial and lateral tibial plateaus separately and the intercondylar eminence width were measured. Finally, the medial and lateral tibial spine height was measured in the PD direction.

The exact definition of the 19 used sizes and distances can be found in the “Appendix” (Table 2) of this article and is the same as described previously [24, 32].

### Statistical analysis

All data analyses for the landmark-based analysis and medial compartment degeneration data were performed using IBM SPSS Statistics for Windows (Version 24.0, IBM Corp., Armonk, NY). To evaluate the presence of medial compartment degeneration in the SMC versus the control group, an *X*^2^ goodness-of-fit test was performed. The rescaling factor and the morphometric measurements of the control group and the SMC group are reported as median and range. A Mann–Whitney *U* test was used to compare the morphological measurements in the SMC group with the data in the control group. To facilitate interpretation of the parameters, the mean results of the SMC group are also converted to a percentage with respect to the mean results of the control group.

For all statistical tests, a *p*-value of less than 0.05 was defined as statistically significant.

Sample size calculation for the Mann–Whitney *U* test was performed in G*Power (version 3.1.9.7, Universität Kiel, Germany) [11] based on an effect size of 1.0, a significance level alpha of 0.05 and power of 0.85. The resulting minimal sample size for equally sized groups was 16 subjects per group.

### Global shape analysis

**Fig. 2:**
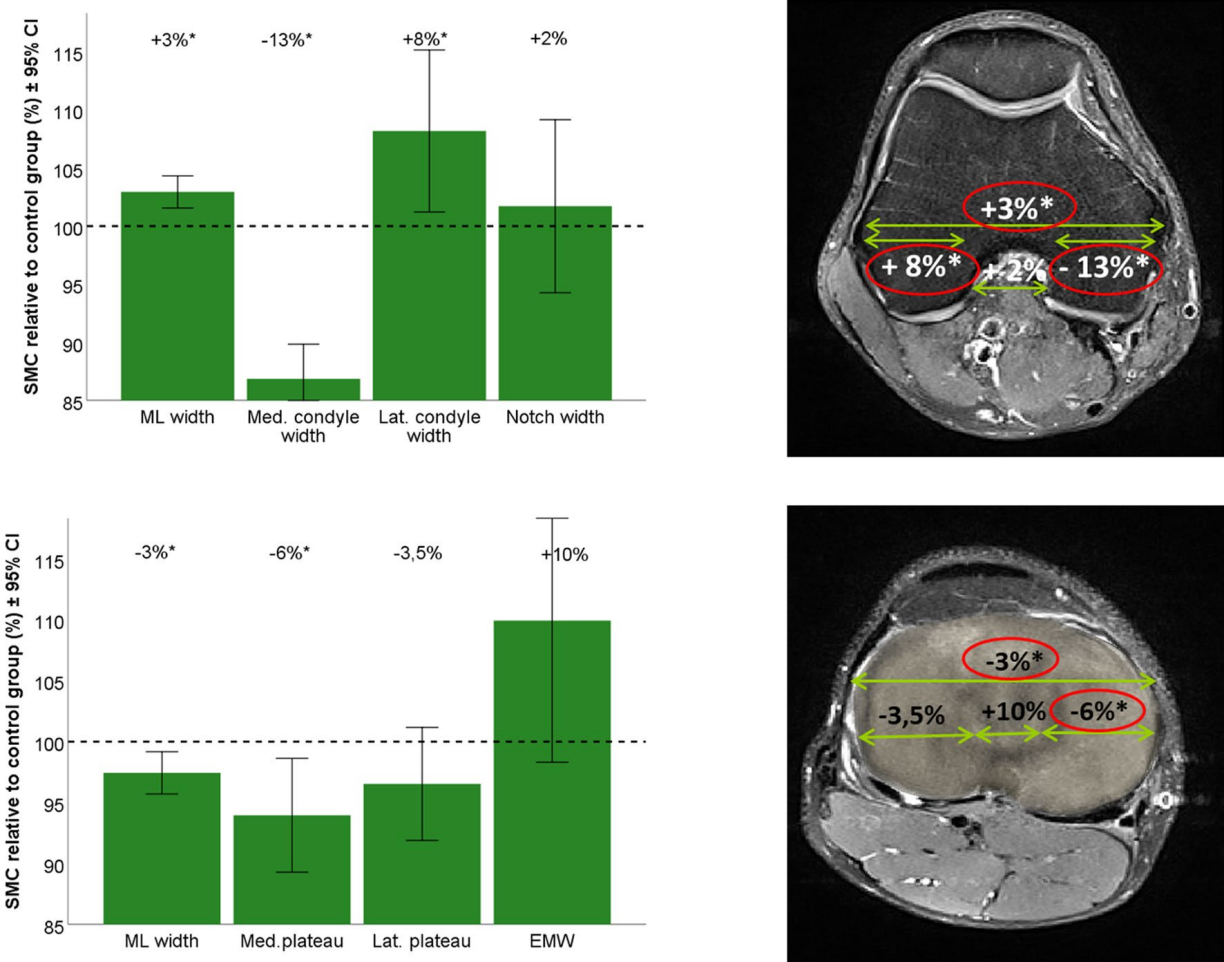
(Left) Graph of the mean SMC group ML measurements in relation to the mean control group measurements (dashed line) for femur (top) and tibia (bottom). (Right) Visualization the mean differences for femur (top) and tibia (bottom) on an axial cross-section of a random subject MRI.

**Fig. 3:**
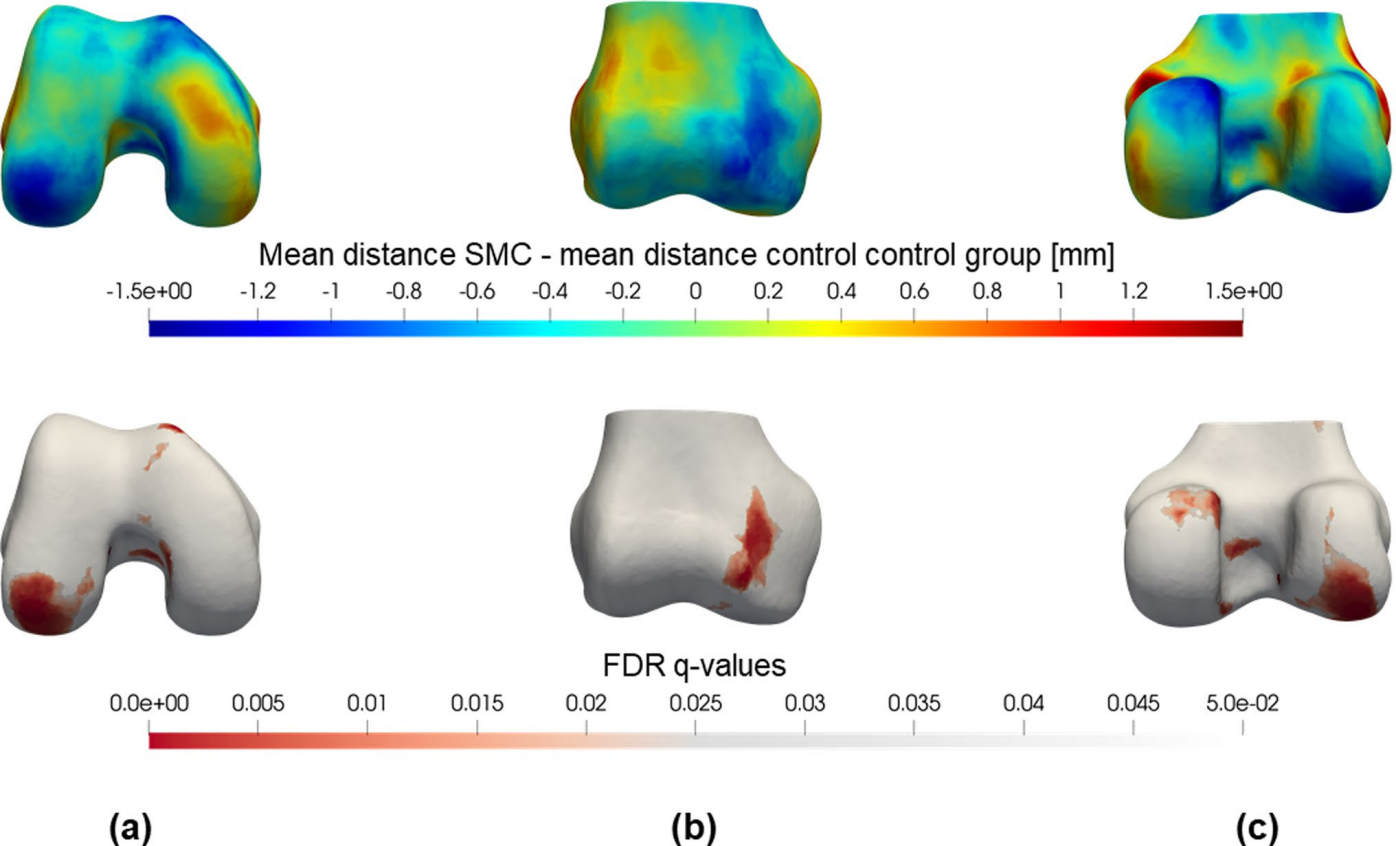
(Upper part) Colour map of the mean femoral shape differences between the SMC and control group. The difference between the mean distances is plotted on the reference femur 3D model (right knee). Blue regions indicate that the SMC femur is on average smaller at that specific location. Conversely, if the SMC femur was on average larger (e.g. caused by a bump or protrusion) the region is coloured red. (Lower part) FDR *q*-values for a permutation *t*-test on femoral shape differences. Distances to the reference femur are compared between the two groups by means of a permutation *t*-test with 1000 permutations and corrected for false discoveries. Only *q*-values < 0.05 were plotted in red on the reference femur (right knee). **a** Distal view; **b** anterior view; **c** posterior view

The global shape analysis aims at finding local shape differences, based on the location of each surface point on the femur and tibia. This analysis consisted of three steps: (1) Find the corresponding points (for every surface, the coordinates that correspond with the same anatomical location are determined) between the individual 3D bone models and a reference 3D bone model of femur and tibia. This process is called the registration of the surfaces and was performed by implementing the iterative process described by Danckaers et al. [6]. Basically, a reference bone shape is elastically deformed to match each respective bone model (femur or tibia) from the dataset. This process was performed twice: first with an arbitrary chosen reference bone shape, the second time with the mean bone shape (constructed by calculating the mean coordinates for all corresponding points) as a reference. (2) Each registered femur and tibia were separately mirrored (left knee shapes were mirrored to match right knee shapes), rotated, shifted and isotropically rescaled to the reference femur or tibia shape, based on the correspondences of all surface points that were found in step 1. This is done by a generalized Procrustes analysis, which has the effect of minimizing the distance between all corresponding surface points. (3) Compare each individual femur and tibia 3D model to the reference bone shape by calculating the pointwise distances between them. The mean bone shape of the control group served as the reference bone shape. The perpendicular distances from the reference shape were compared between the groups and shown in a colour map, plotted on the reference shape (Figs. 2 and 3, upper part). If there are no local shape differences, the distances will be equal to zero (indicated in green on the colour map). If the SMC bone models are on average smaller at that specific location, distances will be negative (indicated in blue on the colour map). Conversely, if the SMC bone model was on average larger, the distances will be positive (and indicated in red on the colour map).

Statistical analysis of these distances was performed to detect differences between the SMC group and the control group. A permutation student *t*-test was done with correction for multiple comparisons (False Discovery Rate, FDR) to avoid type I errors due to the multiple tests [18]. The statistical significance level was defined at a *q*-value of less than 0.05. The significant FDR *q*-values were then mapped in red on the reference bone shape (Figs. 2 and 3, lower part).

## Results

### Medial compartment degeneration

All SMC patients and none of the control group patients showed medial compartment degeneration. The association between knee type and the presence of medial compartment degeneration was significant, *χ*^2^ = 32 (*p* < 0.001). Hence, small medial condyle knees are more likely to develop medial compartment degeneration.

### Landmark-based analysis

The knees in the control group (*N* = 16) were rescaled with a median rescaling factor of 0.98 (min. 0.86; max 1.08), and the knees in the SMC group (*N* = 16) with a median rescaling factor of 0.94 (min. 0.80; max 1.06). This difference was statistically significant (*p* = 0.039). The difference between the smallest (0.80) and largest (1.08) rescaling factor in the total study population (*N* = 32) was 35%. The morphometric measurements for both femur and tibia are summarized in Table 2. The main findings were mediolaterally a smaller medial femoral condyle, a wider lateral femoral condyle, and a wider total distal femur on a smaller tibial plateau. Additionally, both the medial and lateral femoral condyle where anteroposteriorly smaller, as well as the medial tibial plateau and total tibial plateau.

**Table 1:** Overview of the morphometric measurements for the femur and the tibia

Femur				Tibia			
Morphometric parameters femur [mm]	Median control (range)	Median small medial condyle (range)	*p*-value	Morphometric parameters tibia [mm]	Median control (range)	Median small medial condyle (range)	*p*-value
*AP depth*							
				Tibial plateau	52 (49–58)	50 (44–53)	0.001
Medial condyle	64 (60–67)	62 (58–65)	0.001	Medial tibial plateau	46 (43–49)	43 (41–49)	0.026
Medial posterior condyle	28 (23–30)	27 (20–33)	n.s	Medial spine AP position	20 (15–26)	22 (18–26)	n.s
Lateral condyle	66 (63–69)	65 (61–68)	0.023	Lateral tibial plateau	36 (31–43)	39 (34–42)	n.s
Lateral posterior condyle	25 (23–29)	24 (20–29)	0.080	Lateral spine AP position	18 (15–23)	18 (14–24)	n.s
Distal femur	78 (76–82)	82 (77–85)	0.003	Tibial plateau	76 (71–80)	74 (70–78)	0.026
Medial condyle	24 (21–29)	21 (18–23)	< 0.001	Medial tibial plateau	31 (28–34)	29 (27–36)	0.007
Lateral condyle	25 (23–29)	28 (21–35)	0.023	Lateral tibial plateau	33 (29–37)	31 (27–40)	n.s
Notch width	21 (16–26)	21 (15–27)	n.s	Intercondylar eminence width	11 (9–15)	12 (8–16)	n.s
				Medial tibial spine	8 (6–10)	9 (7–13)	0.019
				Lateral tibial spine	7 (4–9)	7 (4–9)	n.s

### Global shape analysis

**Fig. 4:**
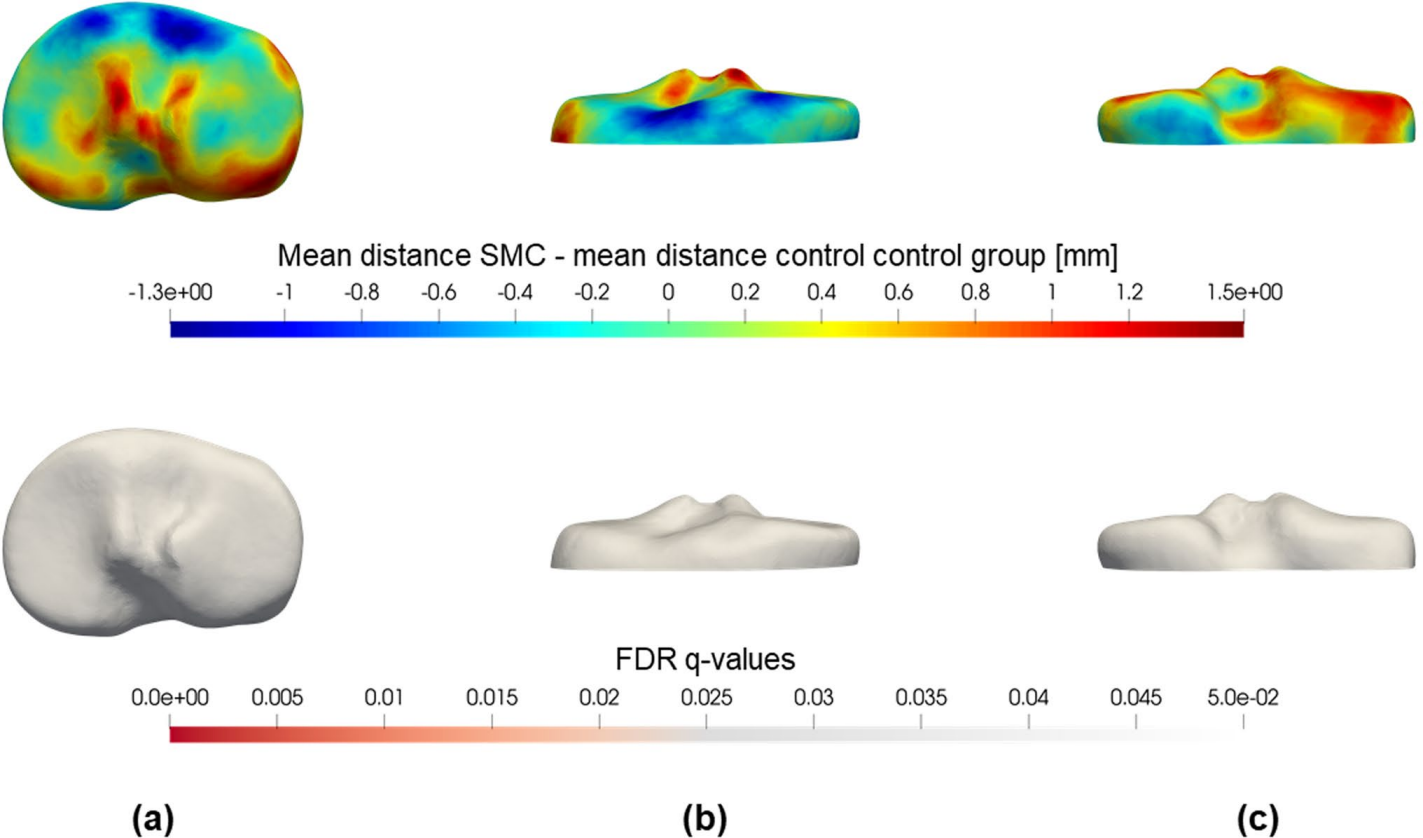
(Upper part) Colour map of the mean tibial shape differences between the SMC and control group. The difference between the mean distances is plotted on the reference tibia 3D model. Blue regions indicate that the SMC tibia is on average smaller at that specific location. Conversely, if the SMC tibia was on average larger (e.g. caused by a bump or protrusion) the region is coloured red. (Lower part) FDR *q*-values for a permutation *t*-test on tibial shape differences. Distances to the reference tibia are compared between the two groups by means of a permutation *t*-test with 1000 permutations and corrected for false discoveries. Only *q*-values < 0.05 were plotted in red on the reference tibia. **a** Proximal view; **b** anterior view; **c** posterior view

The results of the global shape analysis of the femur are colour-plotted in Fig. 3 and all observed trends confirm the results from the landmark-based analysis. The most important significant difference is situated at the inner side of the medial condyle, where the SMC femur is on average 1.1 mm smaller (average FDR *q* = 0.05) with respect to the mean control femur. The SMC knee showed no statistically significant larger or protruding regions. For the tibia, no significant results were detected (Fig. 4).

**Fig. 5:**
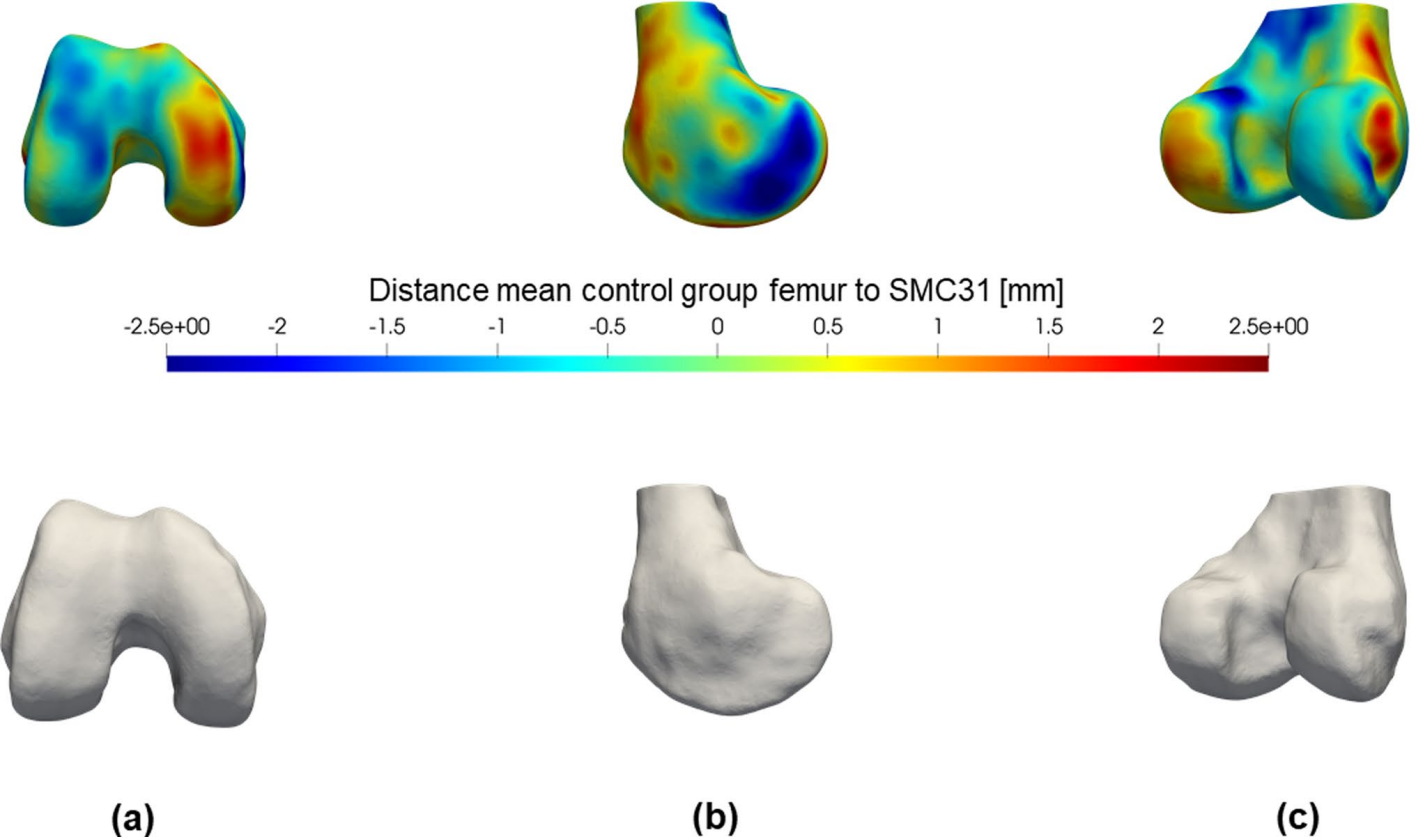
(Upper part) Colour map of the differences between a right SMC femur and the mean control femur. The distances are plotted on the reference femur 3D model. Blue regions indicate that the SMC femur is smaller at that specific location. Conversely, if the SMC femur was larger (e.g. caused by a bump or protrusion) the region is coloured red. (Lower part) Example of an SMC femur case. Visualisation of the case used to calculate the distances from the reference femur in the upper part of this image. **a** Distal view; **b** medial view; **c** posterolateral view

**Fig. 6:**
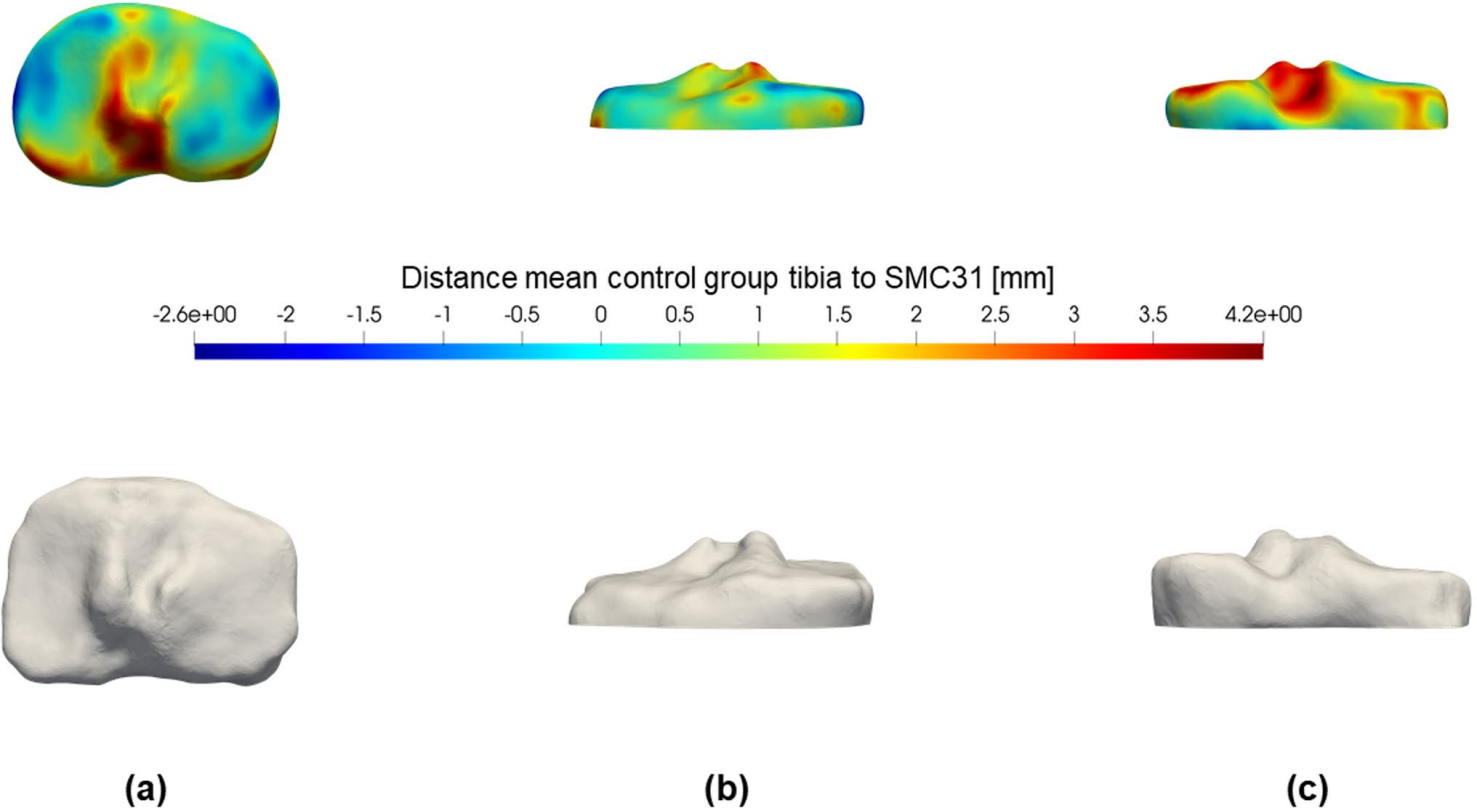
(Upper part) Colour map of the differences between a right SMC tibia and the mean control tibia. The distances are plotted on the reference tibia 3D model. Blue regions indicate that the SMC tibia is smaller at that specific location. Conversely, if the SMC tibia was larger (e.g. caused by a bump or protrusion) the region is coloured red. (Lower part) Example of an SMC tibia case. Visualisation of the case used to calculate the distances from the reference tibia in the upper part of this image. **a** Proximal view; **b** anterior view; **c** posterior view

The extreme case shown in Fig. 5, shows that the combined difference of inner (1.7 mm smaller) and outer side (2.3 mm smaller) of the medial condyle can go up to 4.0 mm (− 17% of the average medial condyle width in the control group). The tibia from that same case (Fig. 6) showed a 2.1 mm higher lateral spine (+ 26% of average medial spine height in control group) and 3.5 mm higher medial spine (+ 50% of average lateral spine height in control group).

## Discussion

The most important finding of this study is the evidence of distinct morphological differences between the control group knee and the small medial condyle knee, which was demonstrated by applying a validated semi-automated landmark-based analysis and a complementary global shape analysis. In the medial compartment, there was a smaller femoral condyle AP and ML, complemented with a smaller tibial plateau ML. However, morphological differences were not limited to the medial compartment, but were also observed at the lateral side. Another remarkable finding was that the overall distal femur was on average wider, while the overall tibial plateau was smaller in the SMC group, which could be an indication for a size and/or shape mismatch between the femoral and tibial geometry.

The subjects included in the SMC group were selected by a senior orthopaedic surgeon, based on observations on the MRI images. The patients were not selected based on their medical records, yet it was observed that all patients consulted the surgeon for complaints related to the medial compartment degenerative tearing. As a result of the *χ*^2^-test, the SMC knees in this study have a higher risk to develop OA. Though this study did not investigate a causal relation between shape and pathology, nor the incidence of this shape variation, this study might be the first step in exploring the link between bone morphology and early degeneration in the medial compartment.

The selection of the SMC cases was performed based on a two-dimensional (2D) MRI slices, hence dependent on the knee position during image acquisition. In fact, this dependence indicates the need for more advanced 3D like those used in this paper. Although the experience based selection was performed on 2D images, the 3D shape analysis results showed the expected shape difference with respect to the ML width of the medial femoral condyle. As this is an exploratory study, the findings can be used in the future to select knee shapes based on a 3D reality rather than a subjective interpretation of the 2D reconstructions.

The underlying mechanism behind the relation between specific morphotypes and pathology has already been investigated for other morphological variations, such as trochlear dysplasia, small notch width and tibia slope. Disturbed biomechanics [1, 4, 7, 12] induce supraphysiological peak stresses and strains [31], which promote early degeneration [19].

A better understanding of the biomechanical implications posed by these distinct morphotypes may help to find the requirements of an optimal therapy strategy for each subgroup of patients. Morphotype is an intrinsic non-modifiable factor in a multifactorial pathogenesis. Especially in high-risk morphotypes modifiable load-related risk factors, such as body mass and certain activities, could be addressed first in a joint-preserving treatment approach of these pathologies. If the total load reduction proves to be unsatisfactory, local supraphysiological contact stress can be decreased further by unloader braces or a corrective osteotomy in a next stage [21]. Also, in end-stage OA patients with a distinctive morphotype, a morphotype-specific total knee prosthesis might be required. For example, knees with trochlear dysplasia (TD) are often more narrow in the ML direction [24, 32]. Therefore, a narrow variant of a total knee prosthesis may be needed to avoid ML overhang.

The current study, which describes the small medial femoral condyle knee as a distinct morphotype, might be related to meniscal pathology. Even when treated according to the gold standards, a meniscal lesion is often associated with the early development of knee OA [3, 10]. Whereas the causal mechanism in the pathogenesis of traumatic tears is clear, degenerative tearing is a multifactorial process. First, genetic predisposition can alter the quality of the meniscal tissue and cartilage [5]. Second, overload due to obesity [9, 17], a high activity level [30] or malalignment [22, 27] (varus or valgus knees) are identified risk factors for early-onset and progression of the degenerative process. In addition to these risk factors, the small medial femoral condyle knee might also be a risk factor leading to overload because of smaller contact areas at the medial compartment.

There is evidence that an arthroscopic partial meniscectomy for meniscus lesions might induce adverse biomechanical effects in a yet indefinite subgroup of patients [25, 35]. Based on personal clinical observation of the senior author (PV), the outcome of a medial partial meniscectomy in an SMC knee is characterized by a higher incidence of rapid progressive degeneration, subchondral insufficiency fractures and massive OA in the medial compartment. The decrease in tibiofemoral contact area [35] caused by the removal of meniscus tissue in an SMC knee is most likely associated with a higher increase of contact stress than it would be in a control group knee. This elevated contact stress is then a driving factor for further degeneration of the affected compartment [19, 21, 26]. Further biomechanical and clinical observational studies are necessary to elucidate this mechanism and confirm this hypothesis. Although the knee morphotype may play an important role in the multifactorial degenerative process, it does not explain the complete etiopathogenesis of knee degeneration.

In this study, two complementary methodologies were applied to evaluate a set of predefined parameters and to evaluate the global shape of the knee models. Where the landmark-based analysis is successful in quantifying several clinically relevant parameters and is easy to interpret, the global shape analysis may detect shape differences which were not captured by the first method. Additionally, it allows for a high level of automation, which makes it a suitable approach to analyse a large number of shapes.

For the femur, the results from the global shape analysis were confirmed by the already validated landmark-based 3D analysis. The results of the tibia analysis showed the same trends. However, in contrast with the landmark analysis, the global shape analysis failed to find significantly different regions. This can be explained by the following mechanism, inherent to the two methodologies. The landmark-based analysis looks for differences in certain pre-defined dimensions of the bone. For example, the ML width of the distal femur is the distance between the medial and lateral femoral epicondyle. If the medial epicondyle in the SMC group tends to be located more medially and the lateral epicondyle more laterally, this results in an additive effect for the ML width of the distal femur in the landmark-based analysis, but not so in the global shape analysis.

The main limitations of this study are the small sample size in both groups and the obvious case selection bias, which are intrinsically linked to the very nature of an exploratory study. Most studies of this exploratory design have a limited number of patients to avoid spending too much time in case of a null effect, as the segmentation and analysis process is time-consuming.

As a consequence of the small sample size, the control group might not be completely representative for a normal knee anatomy on population level. As already shown for the coronal alignment phenotype concept, there can be large variations on population level, even in healthy knees [2, 23]. It is highly unlikely that all physiologic shape variance of the knee joint was captured in this small control group. Therefore, future research should not only focus on how the pathology deviates from the normal, but also in acquiring larger datasets that can more accurately characterize normal knee morphology.

Furthermore, the MRI datasets did not capture the full lower limb. The addition of the coronal alignment phenotype concept (variation in applied load direction) to the knee morphotype concept (variation in shape and hence also surface area) would be highly interesting to estimate the contact stress (by definition: perpendicular projection of the force divided by the surface area) distribution in both compartments.

A clear definition of ‘the small medial femoral condyle knee’ is not yet fully established, as for the moment there is no evidence of a threshold in terms of a knee shape at which the resulting joint contact stress is not tolerated well anymore. However, this study demonstrated that the SMC knee can be identified on a clinical MRI. The main value of this article for daily clinical practice lies in creating awareness for this morphotype as a risk and prognostic factor in medial compartment degeneration. Early identification of knees at risk might help to start conservative treatment at an earlier stage by addressing modifiable risk factors, such as body mass and/or certain activities.

## Conclusion

A new morphotype of the knee demonstrated medial compartment degeneration and was differentiated from a healthy control group based on the following characteristics: a smaller medial femoral condyle and medial tibial plateau; a wider lateral femoral condyle and a wider distal femur on a smaller tibial plateau. This pilot study suggests a role for the SMC knee morphotype in the multifactorial process of medial compartment degeneration.

## Appendix

See Table 2.

**Table 2:** Definition of the morphometric measurements [24, 32]

Distance measurement name	From	Definition landmark	To	Definition plane
Femur ML width	FLE	Femoral lateral epi-condyle (most anterior and distal osseous prominence over the lateral aspect of the lateral femoral condyle)	Plane 6	Medial plane: plane trough FME (femoral medial epicondyle), parallel to FAAx and perpendicular to PCL (posterior condylar line)
Medial condyle ML width at tibiofemoral contact level	FMCIPc	Femoral medial condyle internal point: most lateral point of the cartilage of the medial condyle at the tibiofemoral contact level	Plane 10	External medial condyle plane: plane through FMCEP, parallel to the anatomical axis of the femur and perpendicular to the posterior condylar line
Lateral condyle ML width at tibiofemoral contact level	FLCIPc	Femoral lateral condyle internal point: most medial point of the cartilage of the lateral condyle at the tibiofemoral contact level	Plane 12	External lateral condyle plane: plane through FLCEP, parallel to the anatomical axis of the femur and perpendicular to the posterior condylar line
Notch ML width	FMCIP	Femoral medial condyle internal point: most lateral point of the cartilage of the medial condyle in the axial view of the notch	Plane 11	Internal lateral condyle plane: plane through FLCIP, parallel to anatomical axis of the femur and perpendicular to the posterior condylar line
Medial condyle AP	FMTA	Femoral medial trochlea anterior: the most anterior point of the medial trochlea on the 3D model of the femur, aligned along the longitudinal axis of the femur	Plane 3	PCL plane: plane through FMCP and FLCP and parallel to the anatomical axis of the femur
Medial posterior condyle AP	FMCP	Femoral medial condyle posterior (FMCP): the most posterior point of the medial condyle on the 3D model of the femur, aligned along the longitudinal axis of the femur	Plane 2	Posterior sTEA plane: plane tangent to the posterior cortex, parallel to the surgical transepicondylar axis (OF: plane parallel to the surgical transepicondylar axis, parallel to FAAx and through the posterior cortex)
Lateral condyle AP	FLTA	Femoral lateral trochlea anterior: the most anterior point of the lateral trochlea on the 3D model of the femur, aligned along the longitudinal axis of the femur	Plane 3	PCL plane: plane through FMCP and FLCP and parallel to the anatomical axis of the femur
Lateral posterior condyle AP	FLCP	Femoral lateral condyle posterior: the most posterior point of the lateral condyle on the 3D model of the femur, aligned along the longitudinal axis of the femur	Plane 2	Posterior sTEA plane: plane tangent to the posterior cortex, parallel to the surgical transepicondylar axis (OF: plane parallel to the surgical transepicondylar axis, parallel to FAAx and through the posterior cortex)
Tibial plateau ML	TPM	Tibial plateau medial: he most medial point of the tibial plateau on the 3D model of the tibia	Plane 8’	Lateral tibial plane: plane tangent to TPL and perpendicular to plane 1′ and parallel to the estimated anatomical axis of the tibia
Medial tibial plateau ML	TPM	Tibial plateau medial: the most medial point of the tibial plateau on the 3D model of the tibia	Plane 6’	Medial intercondylar eminence plane: plane tangent to TMIE and perpendicular to plane 1′ and parallel to the anatomical axis of the tibia
Lateral tibial plateau ML	TPL	Tibial plateau lateral: the most lateral point of the tibial plateau on the 3D model of the tibia	Plane 7’	Lateral intercondylar eminence plane: plane tangent to TLIE and perpendicular to plane 1′ and parallel to the anatomical axis of the tibia
Intercondylar eminence width ML	TMIE	Tibial medial intercondylar eminence: the most proximal point of the medial intercondylar eminence	Plane 7’	Lateral intercondylar eminence plane: plane tangent to TLIE and perpendicular to plane 1′ and parallel to the anatomical axis of the tibia
Lateral spine AP position	TLIE	Tibial lateral intercondylar eminence: the most proximal point of the medial intercondylar eminence	Plane 1’	Posterior tibial plane: plane tangent to TMPP and TLPP and parallel to the posterior tibial cortex
Medial tibial spine height	TMIE	Tibial medial intercondylar eminence: the most proximal point of the medial intercondylar eminence	Plane 9’	Medial tibial plateau inferior plane: plane tangent to the most inferior point of the medial tibial plateau and perpendicular to the posterior tibial plane (plane 1′) and tibial lateral plane (plane 8′)
Lateral tibial spine height	TLIE	Tibial lateral intercondylar eminence: the most proximal point of the medial intercondylar eminence	Plane 10’	Lateral tibial plateau inferior plane: plane tangent to the most inferior point of the medial tibial plateau and perpendicular to the posterior tibial plane (plane 1′) and tibial lateral plane (plane 8′)

Visualisations of the landmarks and planes can be found in these publications [24, 32]

## Abbreviations

3D: Three-dimensional
ACL: Anterior cruciate ligament
APM: Arthroscopic partial meniscectomy
AP: Anteroposterior(ly)
ML: Mediolateral(ly)
OA: Osteoarthritis
PD: Proximodistal(ly)
SMC: Small medial condyle
TD: Trochlear dysplasia
